# The Hidden Diet: Determining the Distribution of the Threatened Julia Creek Dunnart (*Sminthopsis douglasi*) Using Eastern Barn Owl (*Tyto javanica delicatula*) Pellets

**DOI:** 10.1002/ece3.71617

**Published:** 2025-06-27

**Authors:** Dana A. Lockhart, Joshua J. Bon, Cameron L. Charley, Stephen G. Kearney, Pia Schoenefuss, Emma L. Gray, Andrew M. Baker

**Affiliations:** ^1^ School of Biology and Environmental Science Queensland University of Technology Brisbane Queensland Australia; ^2^ CEREMADE Université Paris Dauphine‐PSL Paris France; ^3^ Bush Heritage Australia Melbourne Victoria Australia; ^4^ Biodiversity and Geosciences Program Queensland Museum South Brisbane Queensland Australia

**Keywords:** Dasyuridae, detection, dietary analysis, Mitchell grass downs, Queensland, small mammal

## Abstract

Approximately 30% of the world's biodiversity has been threatened or driven to extinction since the 1500s, with Australia losing 10% of its endemic terrestrial mammal species in the last 200 years. The Julia Creek dunnart ( *Sminthopsis douglasi* ) is a threatened small mammal endemic to Queensland (Australia) that requires monitoring and protection. However, there is insufficient ecological data to determine its distribution adequately, and the species has only been sporadically caught in live trapping surveys, with no population size estimates. In the present study, eastern barn owl ( *Tyto javanica delicatula* ) pellet analysis was used to assess a range of locations for the presence of the Julia Creek dunnart as a prey species. Owl pellet deposit sites were chosen to encompass areas of high, medium, and low likelihood of Julia Creek dunnart occurrence based on Australian Government habitat models for the species with the goal of better understanding the species' distribution. In the analysis, Julia Creek dunnarts were present at four (of nine) locations, including all high‐likelihood locations, half of the medium‐likelihood locations, and no low‐likelihood locations. This result supported the accuracy of the Australian Government habitat models. Results also demonstrated the importance of the long‐haired rat ( *Rattus villosissimus* ) in the eastern barn owl diet during plague years. The research will assist with prioritizing sites for protection and monitoring of the Julia Creek dunnart. It also adds support to the utility of owl pellet analysis alongside or independent of traditional surveying techniques, such as live trapping, when targeting cryptic small mammal species.

## Graphical abstract

In the present study, eastern barn owl ( *Tyto javanica delicatula* ) pellet analysis was used to assess a range of locations for the presence of the Julia Creek dunnart ( *Sminthopsis douglasi* ) as a prey species. Owl pellet deposit sites were chosen to encompass areas of high, medium, and low likelihood of Julia Creek dunnart occurrence based on Australian Government habitat models for the species with the goal of better understanding the species' distribution. In the analysis, Julia Creek dunnarts were present at four (of nine) locations, including all high‐likelihood locations, half of the medium‐likelihood locations, and no low‐likelihood locations. This research will assist with prioritizing sites for protection and monitoring of the Julia Creek dunnart.

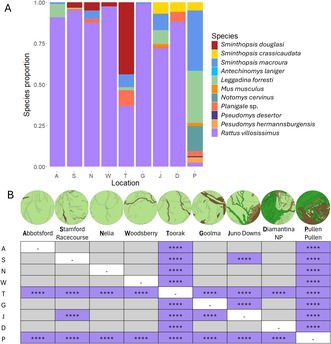

## 1 Introduction

Since the 1500s, approximately 30% of global biodiversity has been threatened or driven to extinction (Isbell et al. 2022). 6%–10% of extinctions have been from Australia, with at least 100 endemic species lost since 1788 (Woinarski et al. 2019). Furthermore, Australia has the highest rate of terrestrial mammal extinctions among all developed nations, having lost over 10% of its 273 endemic terrestrial mammal species in the past two centuries (Woinarski et al. 2015; Waldron et al. 2017). Arid regions of the continent have been particularly affected by severe extinction rates since European settlement, primarily due to invasive predators including the feral cat ( *Felis catus* ) and the European red fox ( *Vulpes vulpes* ) (Morton 1990; Letnic 2000; Woinarski et al. 2015, 2019). Comprehensive monitoring of ecosystems and their species is crucial for effective conservation strategies because inadequate knowledge can result in the misclassification of threatened species and fail to recognize others that require protection (Legge et al. 2018; Moussy et al. 2021). Unfortunately, long‐term ecological monitoring is underfunded, and available resources are only declining (Lindenmayer et al. 2012; Lindenmayer 2017; Lindenmayer and Likens 2018; Ríos‐Saldaña et al. 2018). Conventional methods for surveying wildlife tend to be expensive, labor‐intensive and logistically challenging, especially in arid regions. They also operate on a small spatiotemporal scale and may raise concerns about animal welfare (Perkins et al. 2013; Dundas et al. 2019; Schoenefuss 2023). Thus, there is an urgent need to develop more cost‐effective and less labor‐intensive monitoring techniques that can be adapted to different habitats and scales, ensuring the efficient use of scarce conservation resources (Swan et al. 2013; Dundas et al. 2019; Welbourne et al. 2020; Thomas et al. 2020; Landler and Stefke 2021).

Owl pellet analysis is a method that may overcome these challenges. The vast majority of owls consume their prey whole and subsequently regurgitate pellets that contain identifiable bones, feathers, and scales of vertebrates consumed during their hunts (Morton 1975; Morton et al. 1977; Higgins and Al‐Dabbagh 1999; Hollands 2008). The bones provide a reliable record of prey items, often allowing identification to the species level, particularly for, but not limited to, mammalian craniodental remains (Wallace 1948; Glue 1967; Raczynski and Ruprecht 1974; Higgins and Al‐Dabbagh 1999; Debus and Rose 2007; Bilney et al. 2010; Garcia‐Heras et al. 2017). Eastern barn owls ( *Tyto javanica delicatula* ) are highly suitable for owl pellet analysis in Australian environments. Their hunting range spans a variety of open habitats, including remote arid and semi‐arid regions, which makes them effective detection tools for a range of vertebrate taxa occurring in varied environments (Hyem 1936; Higgins and Al‐Dabbagh 1999). While *T. javanica delicatula* can consume lizards, birds, and invertebrates, they prey primarily on small mammals, making them ideal for detection studies focusing on these prey species (Smith and Cole 1989; Debus et al. 1999, 2008; Palmer 2001; Debus and Rose 2007; Kutt et al. 2020). Owl roosts are usually discovered opportunistically by researchers, allowing for initial clearing of the site and subsequent regular collection of owl pellets. As roost sites are generally located within structures that are partially or fully protected from the elements (e.g., sheds, trees, houses, caves), owl pellets are able to accumulate. Initial collections in arid and semi‐arid Australia have been assumed to contain material up to 5 years old, excluding those considered subfossils (Kutt et al. 2020). However, regular collections after the initial survey can be accurately dated, offering insights concerning present‐day changes in small mammal communities (Schoenefuss et al. 2024), and this is a valuable utility of owl pellet collections (Debus et al. 2008; McDonald et al. 2014; Spencer et al. 2017; Charley et al. 2025a).

Multiple studies have compared the efficacy, cost, and benefits of owl pellet analysis to those of traditional live trapping methods. For example, Heisler et al. (2015) conducted a literature review that assessed 27 studies comparing simultaneous owl pellet analysis and traditional live trapping (including Elliott/Sherman traps, cage traps, and snap traps). Their findings revealed that estimates of small mammal diversity from owl pellets were consistently equal to or greater than those obtained through live trapping. This result was also supported by the recent research of Schoenefuss et al. (2024) in Australia's arid regions. Even for small sample sizes, owl pellet analysis often identifies a higher species richness than live trapping (Torre et al. 2004). Owl pellet analysis is also advantageous as it is non‐invasive (Heisler et al. 2015; Schoenefuss et al. 2024). Live trapping disrupts natural animal behaviors and can induce physiological stress in captured animals (Eccard and Klemme 2013; Waudby et al. 2019).

Furthermore, using owl pellet analysis instead of trapping can lead to significant savings in time and resources (McDonald et al. 2014; Drebet 2022; Schoenefuss 2023). McDonald et al. (2014) reported that owl pellet analysis required only 20% of the time needed for trapping while achieving twice the species richness and sample size. Owl pellet analysis can also effectively mitigate some challenges associated with trapping, such as trap placement, bait selection, and the low abundance, patchy distribution, or trap shyness of target species (Tasker and Dickman 2001; Thompson and Thompson 2007; McDonald et al. 2014; Vieira et al. 2014; Read et al. 2015; Humphrey et al. 2017). *Tyto javanica delicatula* are assumed to hunt in a range up to 10 km from their roosts, allowing for a markedly broader spatial scale of sampling compared to most trapping studies (Hyem 1979; Higgins and Al‐Dabbagh 1999; Perkins et al. 2013; Heisler et al. 2015). They also offer more temporal scope, as owls hunt and deposit pellets throughout the year, whereas trapping typically takes place over a limited duration and is usually restricted to the cooler and drier seasons (Perkins et al. 2013; Heisler et al. 2015; Schoenefuss 2023). Owl pellets cannot provide measurements, population size estimates or tissue samples that require a live animal. However, if the study aims to gather presence/absence information or estimate relative species abundance, then owl pellet analysis is a viable tool.

Some studies have reported preferential hunting behaviors in owls (Smith 1984; Yom‐Tov and Wool 1997; Pavey et al. 2008; Bilney et al. 2010; Garcia‐Heras et al. 2024), including a Hawaiian island barn owl that specializes in hunting native seabirds (Raine et al. 2019). However, most research suggests that barn owls are opportunistic predators, which in theory should offer reliable measures of vertebrate prey presence and abundance (Morton and Martin 1979; Smith and Cole 1989; Yom‐Tov and Wool 1997; Heywood and Pavey 2002; Schoenefuss et al. 2024). For example, Andrade et al. (2016) investigated the hunting behavior of barn owls and produced evidence suggesting they hunt randomly. Such studies support the idea that the proportions of prey species found in owl pellets accurately represent the abundance of those species within their respective communities.

The present study aimed to utilize *T. javanica delicatula* pellet collections to target one of their prey items, a cryptic dasyurid, the Julia Creek dunnart ( *Sminthopsis douglasi* ). *Sminthopsis douglasi* is native to Queensland, Australia and is listed as Vulnerable under state (*Nature Conservation Act* (Qld) 1992) and federal (*Environment Protection and Biodiversity Conservation Act* (Cth) 1999) legislation. The species has an unknown population size (Department of Climate Change, Energy, the Environment and Water 2025), although densities of 0.38 and 0.16 individuals ha^−1^ have recently been estimated for two monitoring sites within Bladensburg National Park, with a mean population estimate comprising 1211 individuals (Bakker, Patterson, et al. 2024). *Sminthopsis douglasi* was first described by Archer (1979) based on just four specimens collected in the downs country of Richmond and Julia Creek between 1911 and 1972. It was considered extinct in the 1980s until Woolley (1992) reported its rediscovery. Since then, *S. douglasi* has been detected at 28 locations (Archer 1976; Woolley 1992; Mifsud 1999, 2000; Kutt 2003). Its known distribution is confined to central‐west and north‐west Queensland, with Lyrian marking the northernmost point, Mount Margaret the westernmost, Moorrinya National Park in the east, and Bladensburg National Park in the south (Woolley 1992; Mifsud 2000, 2001a; Kutt 2003; Baker 2013). Most of the species' known range lies within the Mitchell Grass Downs Bioregion, while Moorrinya National Park is part of the Desert Uplands Bioregion, and Lyrian falls within the Gulf Plains Bioregion. Only two of the locations at which *S. douglasi* has been detected are on protected land, while all others are on private properties primarily used for grazing cattle ( *Bos taurus* ) and/or sheep ( *Ovis aries* ).

*Sminthopsis douglasi* has been only sporadically detected since the early 1990s (primarily using live metal box trapping; Bakker, Patterson, et al. 2024; Bakker, Schoenefuss, et al. 2024). Most records of the species have come from opportunistic findings rather than comprehensive, systematic surveys, leaving the exact limits of its range unclear. Woolley (1992) suggested that *S. douglasi* distribution may be broader than recognized, and subsequently, Kutt (2003) proposed eastern and southern boundaries to the range. However, much of the Mitchell Grass Downs Bioregion is dominated by Mitchell grasses (*Astrebla* spp.), Flinders grasses (*Iseilema* spp.), and cracking clay soils, which are purported habitat preferences of *S. douglasi* (Mifsud 1999; Kutt 2003; Department of Climate Change, Energy, the Environment and Water 2008; Department of Environment and Resource Management 2009; Waudby and Petit 2017; Woolley 2017). This implies that the species' range may extend even further than Kutt (2003) initially proposed. However, many existing records are outdated (being over 20 years old), and given environmental changes due to both climate and land use (Department of Environment and Resource Management 2009; Murphy et al. 2012; Neldner et al. 2017) and recent extreme flooding to the north of the species' known range (Bureau of Meteorology 2019), it is uncertain if *S. douglasi* persists in these areas.

An existing Australian Government habitat distribution model (ecological niche model) for *S. douglasi* (Department of Climate Change, Energy, the Environment and Water 2024a) provided a valuable baseline for categorizing sites as high, medium, and low likelihood of species presence. As *T. javanica delicatula* are known predators of the species (Woolley 1992), owl pellet deposits falling both inside and outside of historical occurrence areas permitted an assessment of distribution model likelihoods for *S. douglasi* . Therefore, the primary aim of the present study was to provide presence/absence information for *S. douglasi* at a range of owl pellet sites, encompassing high, medium, and low likelihood habitats for the species, as determined by the Australian Government ecological niche model. The habitat categories from each site were also broadly examined to determine how they might impact the presence of *S. douglasi* . A secondary aim of this study was to inventory and assess differences in the small mammal community among sites and investigate potential reasons for this. This aim was included to inform future studies that may focus on the same properties or broader regions and to better understand how the owl pellet detection method assesses richness across locations. It also permitted an assessment of how other mammal species may interact with *S. douglasi* , which is a recommended action in the national recovery plan for the species (Department of Environment and Resource Management 2009).

## 2 Methods

### 2.1 Owl Pellet Collection and Locations

**FIGURE 1:**
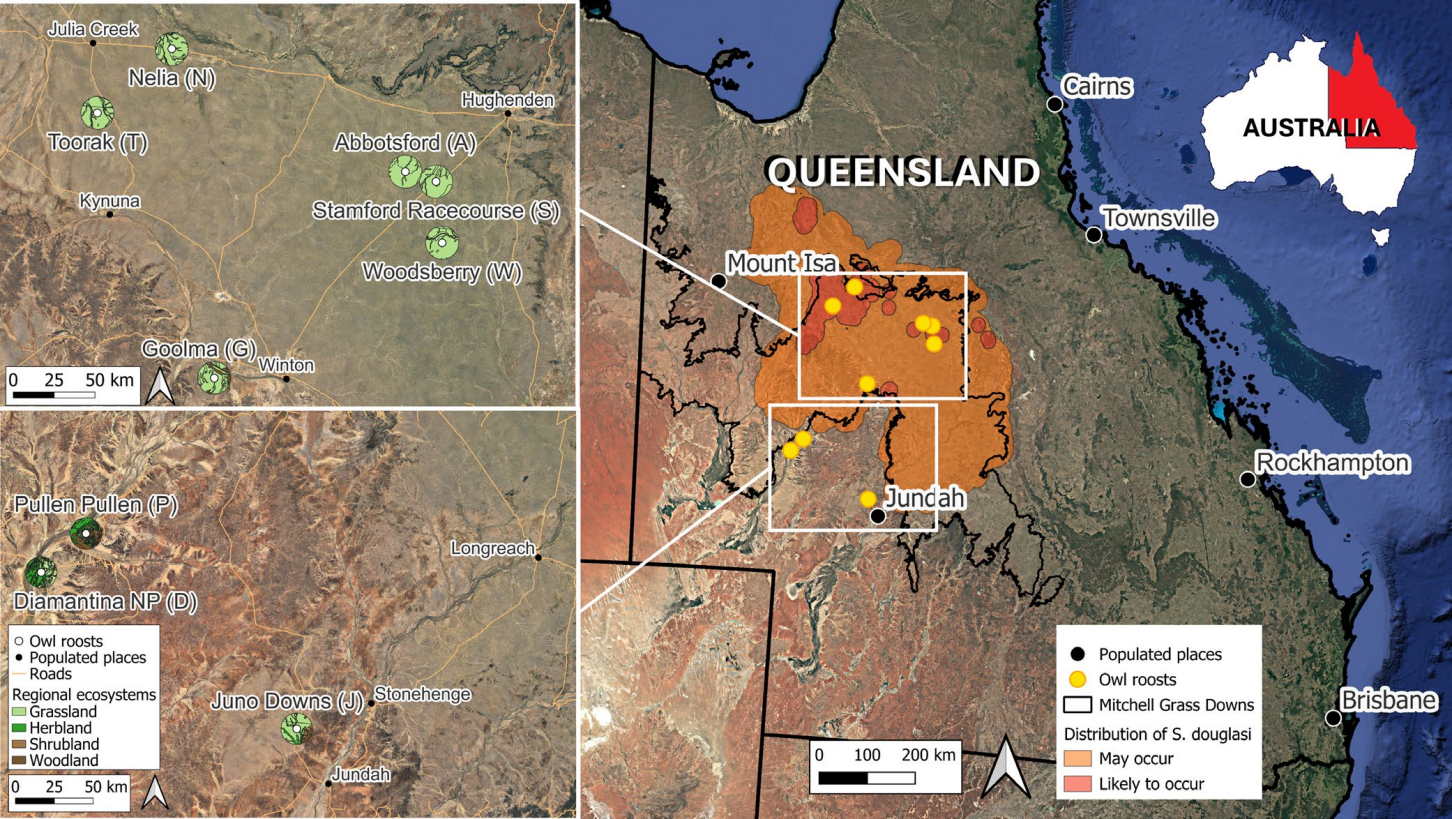
Map of Queensland with white boxes focusing on the owl roosts (white circle) and hunting ranges (assumed 10 km radius from the roost; Hyem 1979) of each owl with the corresponding surrounding habitat categories. Habitat modeling from the Australian Government suggests *Sminthopsis douglasi* may occur in the light orange region and are likely to occur in the dark orange regions (Department of Climate Change, Energy, and the Environment and Water 2024a). The bracketed letter after each owl roost site name refers to their abbreviation in the present study.

The Australian Government habitat distribution model (ecological niche model) for *S. douglasi* (Department of Climate Change, Energy, and the Environment and Water 2024a) was primarily based on observation records, knowledge of habitat preferences and species traits, and provided areas modeled to be high, medium, and low likelihood of *S. douglasi* presence. The attributes considered in forming this model were generic given the lack of knowledge on the species. To evaluate this model, seven owl roost locations were sampled for the presence of *S. douglasi* , as well as the diversity and abundance of the broader small mammal community. These sites included one high‐likelihood location (Nelia), four medium‐likelihood locations (Abbotsford, Stamford Racecourse, Woodsberry and Goolma), and two low‐likelihood locations (Diamantina National Park and Juno Downs) (Figure 1). Between 24 and 100 owl pellets were examined from each site, as dictated by the number of pellets available from each collection (Table 1). Pellet subsets were also taken from each of two owl pellet collections comprising prior published datasets: Toorak (high‐likelihood location; Charley et al. 2025a) and Pullen Pullen Reserve (low‐likelihood location; Kearney et al. 2022; Schoenefuss 2023). One hundred pellets were randomly selected from the initial collection made at Toorak by Charley et al. (2025a), and 98 pellets were included from the initial Pullen Pullen collection and a further two pellets from the second collection that were chosen at random (Kearney et al. 2022; Schoenefuss 2023). Thus, overall, two high‐likelihood locations, four medium‐likelihood locations, and three low‐likelihood locations were analyzed in the present study, comprising a total of nine sites (Figure 1; Table 1). While an even number of roost sites from each likelihood category would have been ideal, site selection was constrained by being able to access properties and locate owl roosts.

**TABLE 1:** Location coordinates and corresponding information from all sites analyzed in the present study.

Location	Coordinates	Bioregion	Subregion	Number of pellets	Date/s of collection	Collection number	Source	Probability
(A) Abbotsford, Stamford	21.322 S, 143.630 E	Mitchell Grass Downs	Central Downs	100	20/04/2024	1st	Present study	Medium
(D) Diamantina National Park	23.752 S, 141.166 E	Channel Country	Goneaway Tableland	24	May – 2009 20/07/2023	1st 2nd	Present study	Low
(G) Goolma, Winton	22.416 S, 142.612 E	Mitchell Grass Downs	Central Downs	100	Late Oct – 2023 19/04/2024	1st 2nd	Present study	Medium
(J) Juno Downs, Jundah	24.535 S, 142.830 E	Channel Country	Goneaway Tableland	100	07/05/2009	2nd	Present study	Low
(N) Nelia, Julia Creek	20.654 S, 142.205 E	Gulf Plains	Woondoola Plains	40	14/04/2023	1st	Present study	High
(S) Stamford Racecourse, Stamford	21.249 S, 143.815 E	Mitchell Grass Downs	Central Downs	100	12/04/2023 03/10/2023 20/04/2024	1st 3rd 4th	Present study	Medium
(T) Toorak, Julia Creek	21.033 S, 141.800 E	Mitchell Grass Downs	Central Downs	100	13/04/2023	1st	Charley et al. (2025a)	High
(P) Pullen Pullen Reserve	CONFIDENTIAL	Channel Country	Southwestern Downs	100	11/08/2019 22/10/2019	1st 2nd	Kearney et al. (2022), Schoenefuss (2023)	Low
(W) Woodsberry, Stamford	21.577 S, 143.884 E	Mitchell Grass Downs	Central Downs	31	30/05/2023 03/10/2023 20/04/2024	1st 2nd 3rd	Present study	Medium

*Note:* Owl pellets were generally collected from roost sites on a 6‐monthly basis. Kutt et al. (2020) assumed that pellets from the first collection could be up to 5 years old.

**FIGURE 2:**
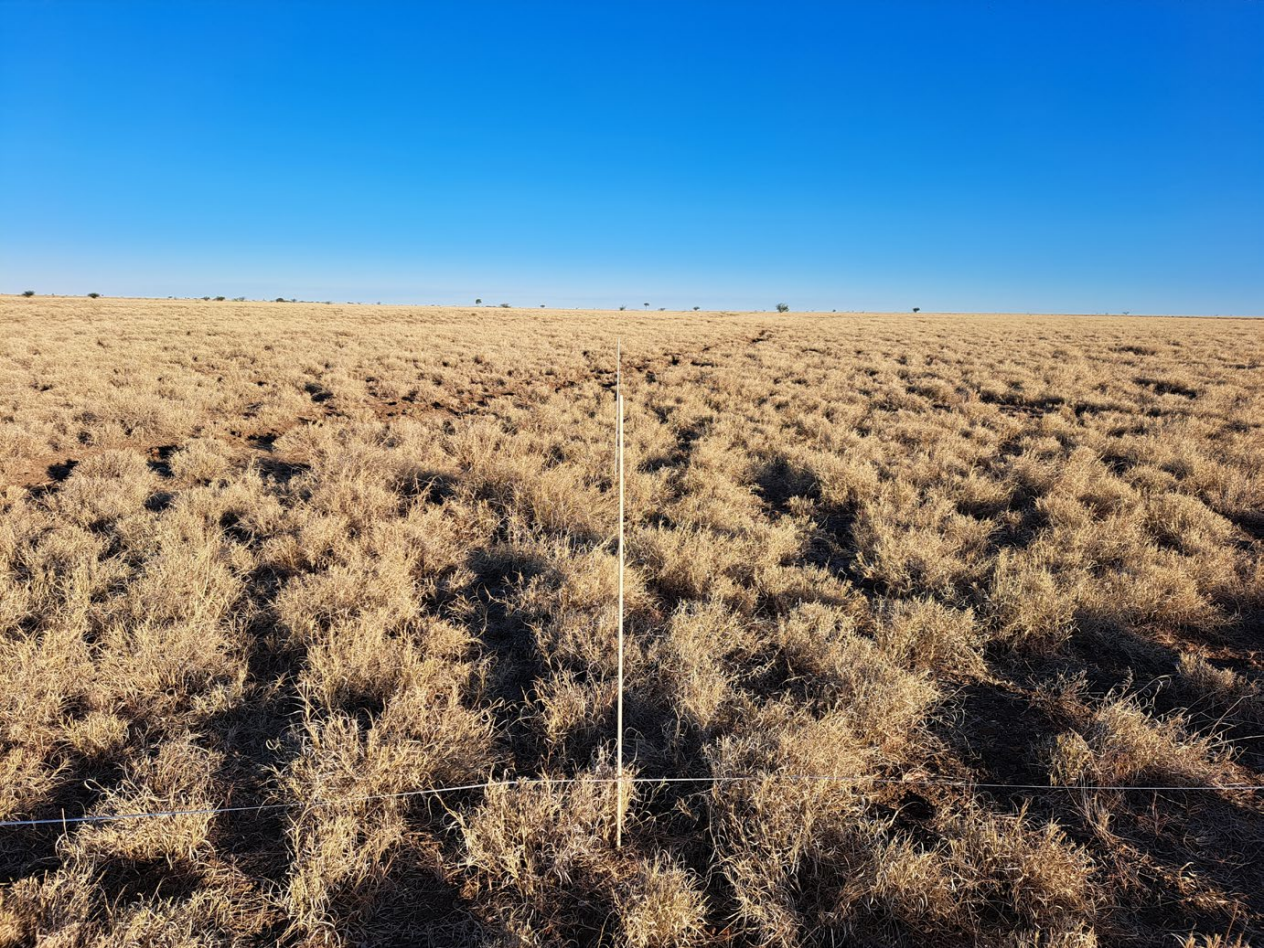
Representative photograph of the Mitchell Grass Downs Bioregion (Woodsberry Lane), displaying Mitchell (*Astrebla* spp.) tussock grasslands on cracking clay soils. Photograph: Emma Gray, June 2024.

The locations analyzed fell within three Bioregions: Mitchell Grass Downs, Channel Country, and Gulf Plains (Table 1). The Mitchell Grass Downs is generally made up of rolling treeless plains consisting of Mitchell (*Astrebla* spp.) tussock grasslands on cracking clay soils with some ridges, rivers, and gorges (Figure 2; Commonwealth of Australia 2008a; Sattler and Williams 1999). The Channel Country is comprised primarily of vast braided, flood and alluvial plains that are encompassed by gravel or gibber plains, dunefields, and low ranges on a range of soil types, including cracking clay (Commonwealth of Australia 2008b; Sattler and Williams 1999). The dominant vegetation groups are *Astrebla* spp. tussock grasslands, gidgee ( *Acacia cambagei* ), and spinifex (*Spinifex* spp.). The southernmost section of the Gulf Plains, subregion Woondoola Plains, is characterized by extensive alluvial plains on red‐brown soils, often with surface gravel (Commonwealth of Australia 2008c; Sattler and Williams 1999). The dominant vegetation types are open woodlands comprised of eucalyptus (*Eucalyptus* spp.) and tea‐tree (*Melaleuca* spp.). The vast majority of land use in these Bioregions is grazing by cattle and sheep (96%, 91%, and 93%, respectively), with Abbotsford, Goolma, Juno Downs, Toorak, and Woodsberry roost sites falling on private property.

Owl pellets processed in the present study were collected from the roost sites approximately every 6 months to limit disturbance of the owls, which may otherwise prevent them from returning to the roost (Table 1). While this does make it possible to date pellets and compare small mammal communities over time, the purpose of the present study was to gather presence/absence data from a range of sites. Thus, the dataset was not appropriate for temporal comparisons. Collected pellets were placed in sealed plastic bags and transported to the Queensland University of Technology (QUT) for dissection. The two external published datasets were also incorporated (Table 1).

### 2.2 Owl Pellet Dissection and Identification

Before all work with owl pellets, authors were vaccinated against Q fever, as a precaution aligning with health and safety protocols. Collected owl pellets were dissected in a QUT laboratory biosafety cabinet using tweezers to separate the bones and hair and placed in two containers. Care was taken with the craniodental material to ensure all fragments were preserved for subsequent identification. Specimen containers were frozen at −20°C for 10 days, as required by the Queensland Museum, to kill any live arthropods that may otherwise infest the collection. Bone specimens were then transported to the Queensland Museum for identification.

Identification of mammal dentaries was completed with reference to registered museum specimens and publications by Archer (1976, 1981), Blacket et al. (2008), and Van Dyck et al. (2013). Hair imprint analysis, while a viable technique, has not been deemed reliable in Australia for accurate identification of mammals to the species level, including for *Sminthopsis* spp. (Lobert et al. 2001). Environmental DNA (eDNA) is another such method that could be used to detect additional prey species within owl pellets (Schoenefuss 2023); however, it was outside the scope of the present study. Birds were identified to the lowest rank possible by comparing beaks and skulls to identified, registered museum specimens and with advice from Queensland Museum curatorial staff. Mammals and birds could be identified to species level in most cases; lizards and frogs were identified to order. No effort was made to identify or enumerate invertebrates.

**FIGURE 3:**
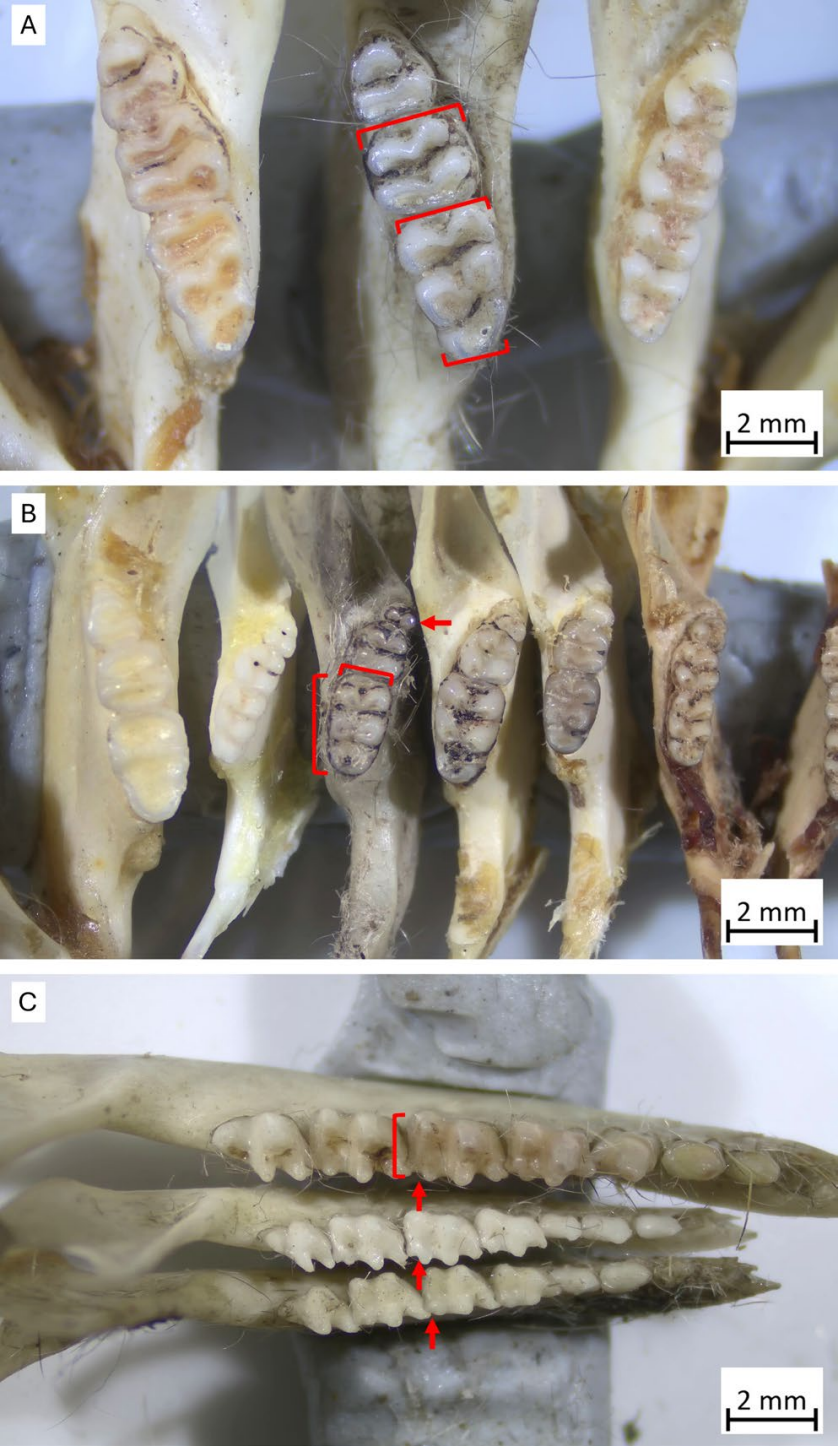
(A) Left to right, molar teeth in dentary of *Rattus villosissimus* (museum specimen: J6723), *R. villosissimus* (owl pellet sample: WG96) and *R. rattus* (museum specimen: J6716). (B) Left to right, molar teeth in dentary of *Notomys fuscus* (museum specimen: J10009), *Pseudomys delicatulus* (museum specimen: JM10912), *Leggadina forresti* (owl pellet sample: JJD91), *L. forresti* (museum specimen: J5113), *L. lakedownensis* (museum specimen: JM18403) and *P. hermannsburgensis* (museum specimen: JM1823). (C) Top to bottom, molar and premolar teeth in dentary of *Sminthopsis douglasi* (owl pellet sample: SRS38), *S. macroura* (owl pellet sample: JJD08) and *S. crassicaudata* (owl pellet sample: JJD08). The hypocristid (ridge) can be seen either connecting or not connecting the entoconid on the M_2_ and M_3_ of each specimen. Red arrows and brackets indicate discriminating sizes, shapes, and features.

For mammals, *Rattus* species were identified by the comparative size (in particular, width) of the molar teeth (Figure 3A). Other rodents were identified based on the shape, size, and angle of emergence of the teeth (Figure 3B). For example, Forrest's mouse ( *Leggadina forresti* ) and Lakeland Downs mouse ( *L. lakedownensis* ) can be distinguished by the latter's shorter length and width of the M_1_. Juvenile rodents were identified by the non‐complete emergence of the M_3_, though they were combined with adults for the present analysis. Dasyurid mammals, specifically dunnarts (*Sminthopsis* spp.), were identified by the size of the teeth and length of the jaw, followed by assessment of the M_2_ and M_3_ hypocristid and whether it contacted the entoconid (Figure 3C) (refer to Archer 1981). In *S. douglasi* and the stripe‐faced dunnart ( *S. macroura* ) the hypocristid does not contact the entoconid, as it does with the fat‐tailed dunnart ( *S. crassicaudata* ; Archer 1981). However, *S. douglasi* is much larger than *S. macroura* , with an enlarged C1 and a tooth row of ≥ 5.6 mm (M^1^‐M^3^), ≥ 11.8 mm (C^1^–M^4^) and ≥ 7.3 mm (M_1_–M_4_) (Baker et al. 2025). Juvenile *S. douglasi* resemble *S. macroura* ; however, juvenile *Sminthopsis* spp. display a characteristic incomplete emergence of the P3, being shorter than or equal to the crown height of the P2. Some juvenile *Sminthopsis* spp. also have an incomplete emergence of the M_4_. Planigales (*Planigale* spp.) were not identified below genus due to species similarities, with spatial and morphological overlap between the long‐tailed planigale ( *P. ingrami* ) and narrow‐nosed planigale ( *P. tenuirostris* ) (Archer 1976; Blacket et al. 2008; but see Charley et al. 2025b).

The number of left and right dentaries was used to calculate the minimum number of individuals within each owl pellet. For example, one left dentary and two right dentaries of a mammal species were classified as two individuals. For bird species, one beak and two skulls were identified as two individuals.

### 2.3 Analyses

High, medium, and low probability locations across the Mitchell Grass Downs were defined according to the Australian Government's habitat distribution model (ecological niche model) for *S. douglasi* (Figure 1 and Table 1; Department of Climate Change, Energy, and the Environment and Water 2024a). The habitat distribution model relies on historical records of *S. douglasi* presence, knowledge of habitat preferences, and species traits. Locations with a high likelihood of *S. douglasi* presence were based on category two—“species or species habitat is likely to occur,” medium likelihood on category one—“species or species habitat may occur,” and low likelihood on areas outside categories one and two. Nelia and Toorak were considered sites with a high probability of *S. douglasi* presence as they are located within category two and have historical records. Abbotsford, Goolma, Stamford Racecourse, and Woodsberry were labeled medium likelihood as they are within category one and *S. douglasi* known range but have no historical records. Low‐likelihood sites (Diamantina National Park, Juno Downs, and Pullen Pullen Reserve) were those outside the known range and categories one and two. The low‐likelihood locations were included here because they occur near the modeled distribution and possess the known generic habitat requirement of cracking clay soils.

Habitats of the nine sites were assessed by clipping the regional ecosystems (Queensland's method of categorizing habitat types; Queensland Government 2025) to the approximate presumed hunting range of *T. javanica delicatula* (10 km radius from the roost site; Hyem 1979; Higgins and Al‐Dabbagh 1999) in QGIS (QGIS Development Team 2024). The habitat types were then categorized and simplified according to their structure code; for example, grassland, herbland, shrubland, and woodland. The regional ecosystem structure code defines the habitat by its overarching Bioregion, dominant soil type, and vegetation genus or species. For example, if the most prevalent vegetation was *Astrebla* spp., the structure code could be open tussock grassland, which would be simplified to grassland for the purpose of the present study. If the habitat was part of a heterogenous polygon (i.e., a polygon containing multiple regional ecosystems), then the structure code of the dominant regional ecosystem was used. The total area of each category was then calculated in QGIS and converted into a proportion of the owl's assumed hunting range. Differences between habitat category compositions were compared using Fisher's Exact Tests between each pair of locations in R (v4.4.1; R Core Team 2024) with Bonferroni adjustments.

Incidence and abundance rarefaction and extrapolation curves for small mammal species were created in R Statistical Software (v4.4.1; R Core Team 2024) using packages iNEXT (Hsieh et al. 2024) based on the Choa et al. (2014) framework. Rarefaction determined the approximate number of owl pellets required to approach the asymptote of species richness (*q* = 0). While rarefaction cannot be extrapolated to infer the actual total species richness of a location (Gotelli and Colwell 2001), qualitative observations may be made. When the curve approaches its asymptote, it may be inferred that the sample size was sufficient (Purger and Szép 2021). Conversely, if the curve continues to rise, it suggests that a larger sample size is needed.

The probability of detection curves were estimated by assuming a binomial distribution for the identification of *S. douglasi* in pellet samples from each site where *S. douglasi* was detected. According to the estimated binomial probability, the probability of detection expected from alternative pellet sample sizes (*n*) was calculated for each site. The binomial model is also used to determine the approximate number of pellets needed to reach a high probability of a single detection (*pn*) of *S. douglasi* (*pn* > 0.99; van Strien et al. 2015; McCullum 2005; Schoenefuss et al. 2024).

An Index of Relative Importance (IRI) was used to rank identifiable vertebrate species by importance in the *T. javanica delicatula* diet. The rank is given by the equation (numerical percentage + volumetric percentage) × frequency of occurrence percentage. The calculation overcomes the biases associated with each factor (detailed in Pinkas et al. 1971; Hart et al. 2002). Body weights of mammals were derived from the *Field Companion to the Mammals of Australia* (Van Dyck et al. 2013) and Kutt et al. (2020). Only mammals and birds were considered in this measurement, as other vertebrates could not be identified to species level. It has been reported that owls and raptors do eat invertebrate species, yet those could also have first been consumed by birds or reptiles, instead of by the owl directly. Thus, we decided not to quantify or identify invertebrates as prey items. IRI was specifically used to allow for in‐depth comparison and meta‐analyses between owl diet studies, whether this be through numerical percentages, frequency of occurrence percentages, or the IRI ranking itself.

Differences between mammal community compositions were compared using Fisher's Exact Tests between each pair of locations in R (v4.4.1; R Core Team 2024) with Bonferroni adjustments. For these tests, all non‐mammalian species were removed from the dataset. Additionally, the Shannon's Diversity Index (Shannon 1948; Spellerberg and Fedor 2003) was used to analyze each site for the dataset including all species (excluding unidentified categories) and for the dataset including only mammals.

## 3 Results

A total of 495 owl pellets were processed in the present study, with a further 200 pellets incorporated from external datasets (100 from Toorak (Charley et al. 2025a) and 100 from Pullen Pullen Reserve (Kearney et al. 2022; Schoenefuss 2023)), for a combined total of 695 pellets assessed. The 495 pellets from the present research took ~120 h to process, for a combined average of ~15 min for dissection and identification of vertebrate prey contents from each pellet. The pellet collection took ~18 h when completed as an addition to a parallel study in the same approximate area (live trapping field work).

### 3.1 Presence of *S. douglasi*

**FIGURE 4:**
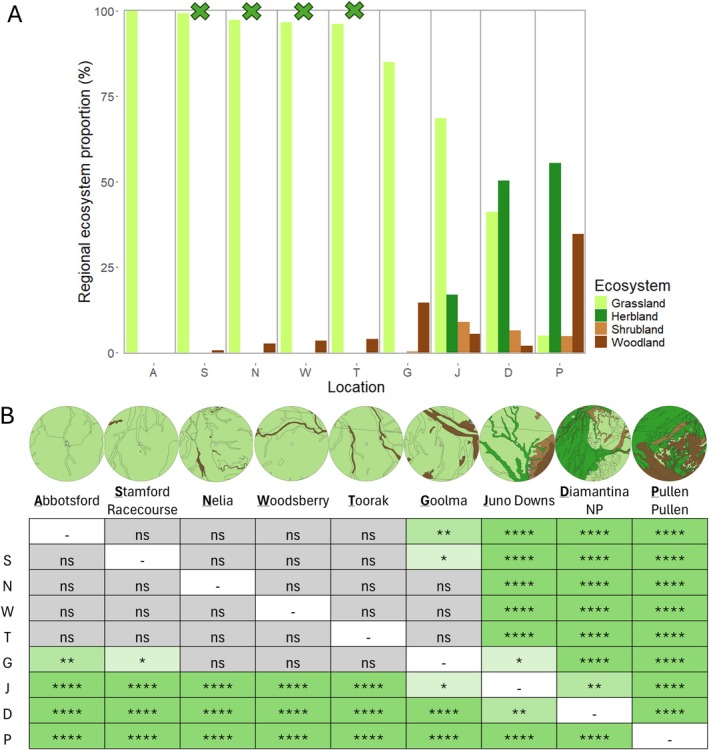
(A) Percentage of habitat categories at the nine sites shown from most grassland to least grassland. The green Xs at the top of the bar chart indicate that *S. douglasi* was present at those locations. (B) Significance of Fisher's Exact Tests after Bonferroni adjustments (represented by the number of * with the maximum being *p* = 7.78 × e^−9^ at **** pairings) between each pair of sites (shown both above and below the diagonal). Circles above each site name show habitat falling within the presumed 10 km owl hunting radius. The habitat color key in (B) is the same as for (A). ns: Not significant.

*Sminthopsis douglasi* specimens were present in owl pellets from two high‐probability sites with historical records (Nelia [2 individuals from 40 pellets] and Toorak [36 individuals from 100 pellets]) and two medium probability sites with no previous records of *S. douglasi* (Woodsberry [1 individual from 31 pellets] and Stamford Racecourse [4 individuals from 100 pellets]). All sites with *S. douglasi* present were within the known distributional range boundary for the species. Overall, both high‐likelihood sites supported *S. douglasi* , as well as two (of four) medium‐likelihood locations, and no (of three) low‐likelihood locations. The proportion of *S. douglasi* in prey items at each of these sites, most to least, was 33.1% from Toorak, 3.8% from Nelia, 2.9% from Stamford Racecourse, and 2.3% from Woodsberry. Fisher's Exact Tests showed that the habitat categories surrounding owl roosts at Diamantina National Park, Pullen Pullen Reserve, and Juno Downs significantly differed from all other sites (*p* < 0.0013). Their difference was primarily based on the percentage of grassland, with Pullen Pullen having the lowest proportion (5.0%), Diamantina National Park having the second lowest (41.2%), and Juno Downs having the third lowest (68.6%; Figure 4A). Pullen Pullen and Diamantina National Park also had the highest proportions of herbland, with 55.5% and 50.3%, respectively. Of the five locations with the highest proportion of grassland, *S. douglasi* was present at four. However, no *S. douglasi* was found at Abbotsford (100 pellets), even though it had the highest proportion of grassland (100%) and was adjacent to Stamford Racecourse (which supported the species).

**FIGURE 5:**
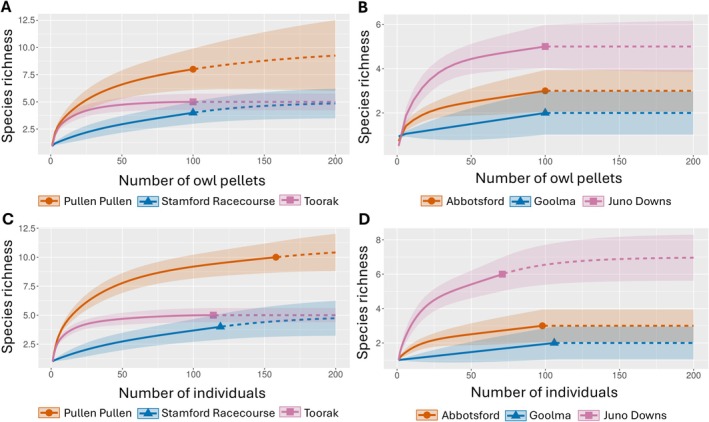
(A) and (B) Incidence rarefaction of the six sites' small mammal species richness, which have a sample size of 100 owl pellets. (C) and (D) Abundance rarefaction of the six sites' small mammal species richness. Extrapolation was performed to a sample size of 200 to capture the predicted plateau or rise in species richness.

The incidence rarefaction curves of Toorak, Abbotsford, Goolma, and Juno Downs all leveled off close to the curve asymptotes at the 100‐pellet sample size (Figure 5A,B). Stamford Racecourse and Pullen Pullen Reserve species richness was predicted to rise further beyond the sample size surveyed. Similar results were present when adjusted for abundance rarefaction curves (Figure 5C,D). Both Stamford Racecourse and Pullen Pullen species richness was predicted to rise given more individuals were identified. However, Juno Downs species richness was also predicted to rise with more individuals identified. Additionally, Pullen Pullen had the highest mammal species richness, with eight mammal species identified, followed by Toorak and Abbotsford, with a mammal species richness of five.

**FIGURE 6:**
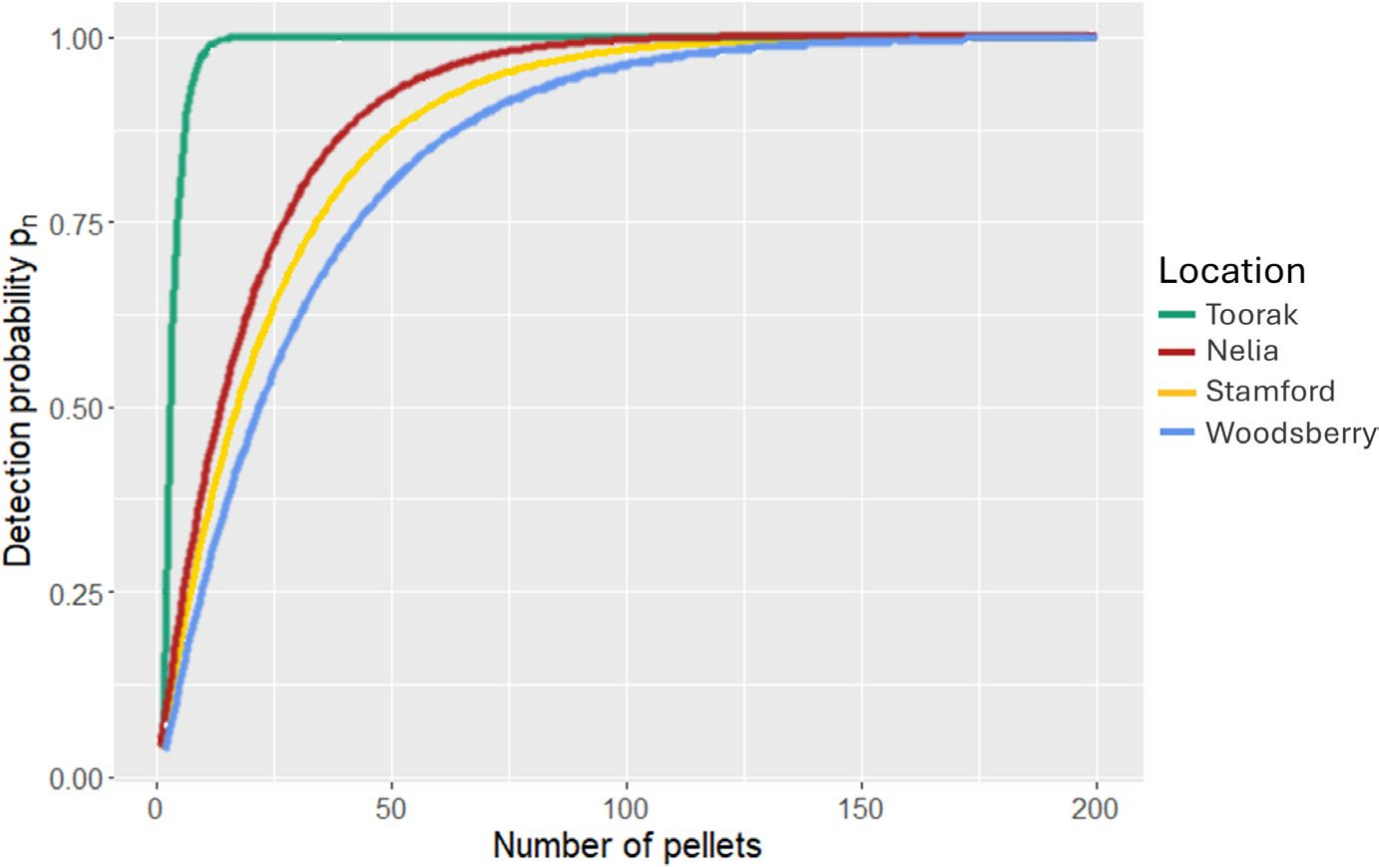
Probability of detection curves for *S. douglasi* at each site where they were present. Curves were extrapolated to a sample size of 200 to capture the rise and following plateau. Actual sample sizes were 100 from Toorak, 40 from Nelia, 100 from Stamford, and 31 from Woodsberry.

The probability of detection curves showed that *S. douglasi* could be confidently detected (*pn* > 0.99) after 10 pellets from Toorak, which was markedly earlier than the other sites where it was present (Figure 6). At Nelia, the species could be confidently detected after 75 pellets, followed by Stamford (100) and Woodsberry (125) (Figure 6).

### 3.2 Differences Between Sites

**FIGURE 7:**
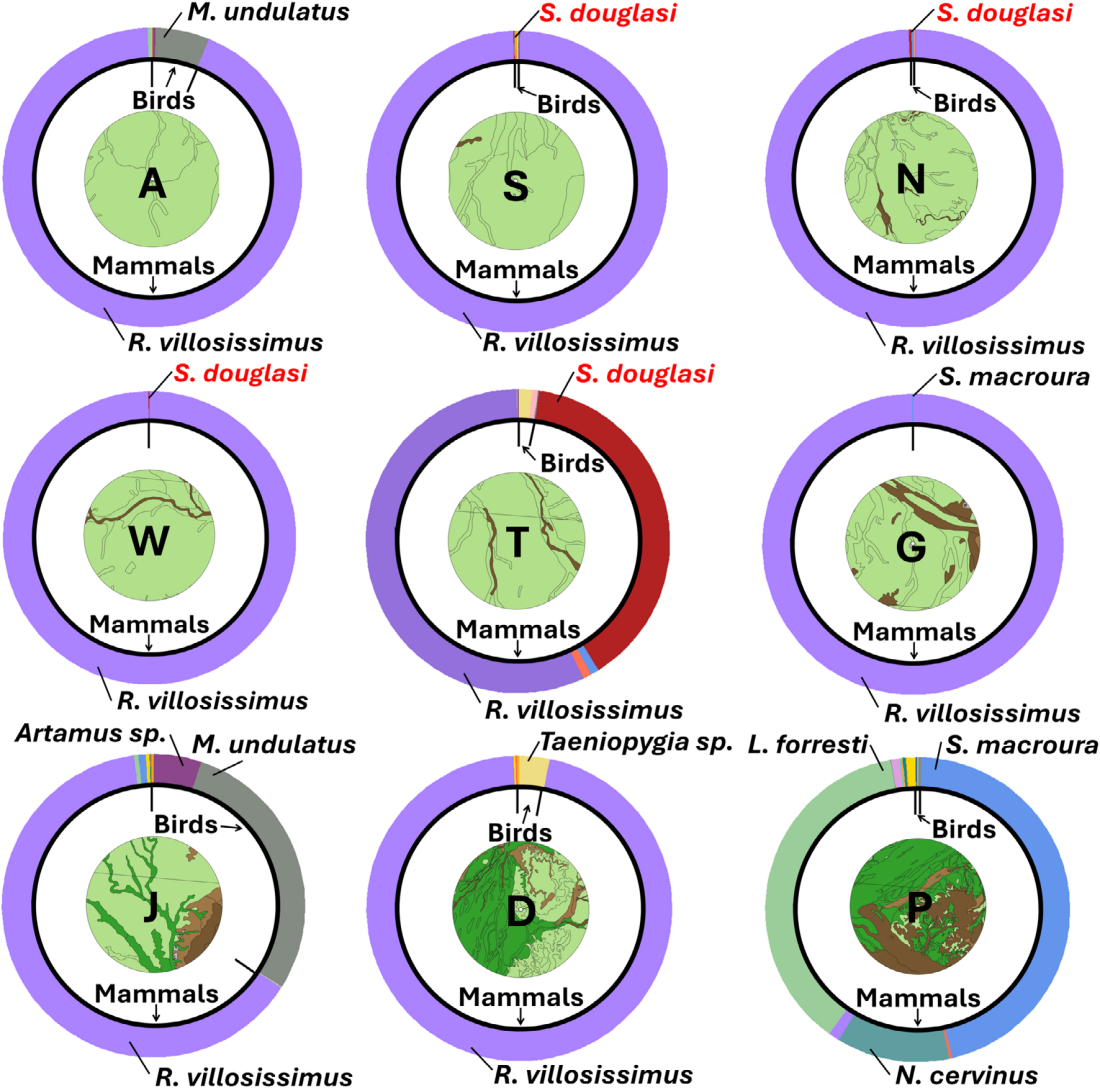
Index of Relative importance proportion at each of the nine sites. The center circle displays the habitat types at each location (light green: Grassland; dark green: Herbland; light brown: Shrubland; dark brown: Woodland) within the assumed 10 km hunting radius of the eastern barn owl ( *Tyto javanica delicatula* ). The inner (thin, black) ring demonstrates the proportion of mammals to birds, demarcated by black strokes. The outer (thick, colored) ring demonstrates the proportion of different species, with important species labeled. A: Abbotsford; S: Stamford Racecourse; Nelia: Nelia; W: Woodsberry; T: Toorak; G: Goolma; J: Juno Downs; D: Diamantina National Park; P: Pullen Pullen Reserve.

The vertebrate Index of Relative Importance (IRI) calculations (Appendix S1) for eight of nine locations indicated that the long‐haired rat ( *Rattus villosissimus* ) was the most important prey item in *T. javanica delicatula* diet, excluding Pullen Pullen Reserve (Figure 7). At Stamford Racecourse, *S. douglasi* only comprised 0.1% of the total IRI (four individuals). *Sminthopsis douglasi* was also present at Nelia, where it comprised 0.3% of the total IRI. Only two species were found at Woodsberry, *R. villosissimus* , and *S. douglasi* , representing > 99.9% and < 0.1% of the total IRI, respectively (Figure 7). Similarly, at Goolma, only two species were identified, with *R. villosissimus* comprising > 99.9% of the total IRI at that site, and only a single stripe‐faced dunnart ( *S. macroura* ) was identified. At Abbotsford, the second most important prey item was a bird, the budgerigar ( *Melopsittacus undulatus* ; 5.8%). At Diamantina National Park, the second most important prey item was also a bird, the finch (*Taeniopygia* sp.; 3.2%). Juno Downs was somewhat different, given the high secondary importance of *M. undulatus* (28.9%). However, the two most unique sites were Pullen Pullen Reserve and Toorak. At Pullen Pullen, *S. macroura* was the most important prey item (46.0% of the IRI), and second was the native mouse, *Leggadina forresti* (37.3%); *R. villosissimus* only comprised 1.2% (Figure 7). At Toorak, *S. douglasi* was the second most important prey item (38.8%).

**FIGURE 8:**
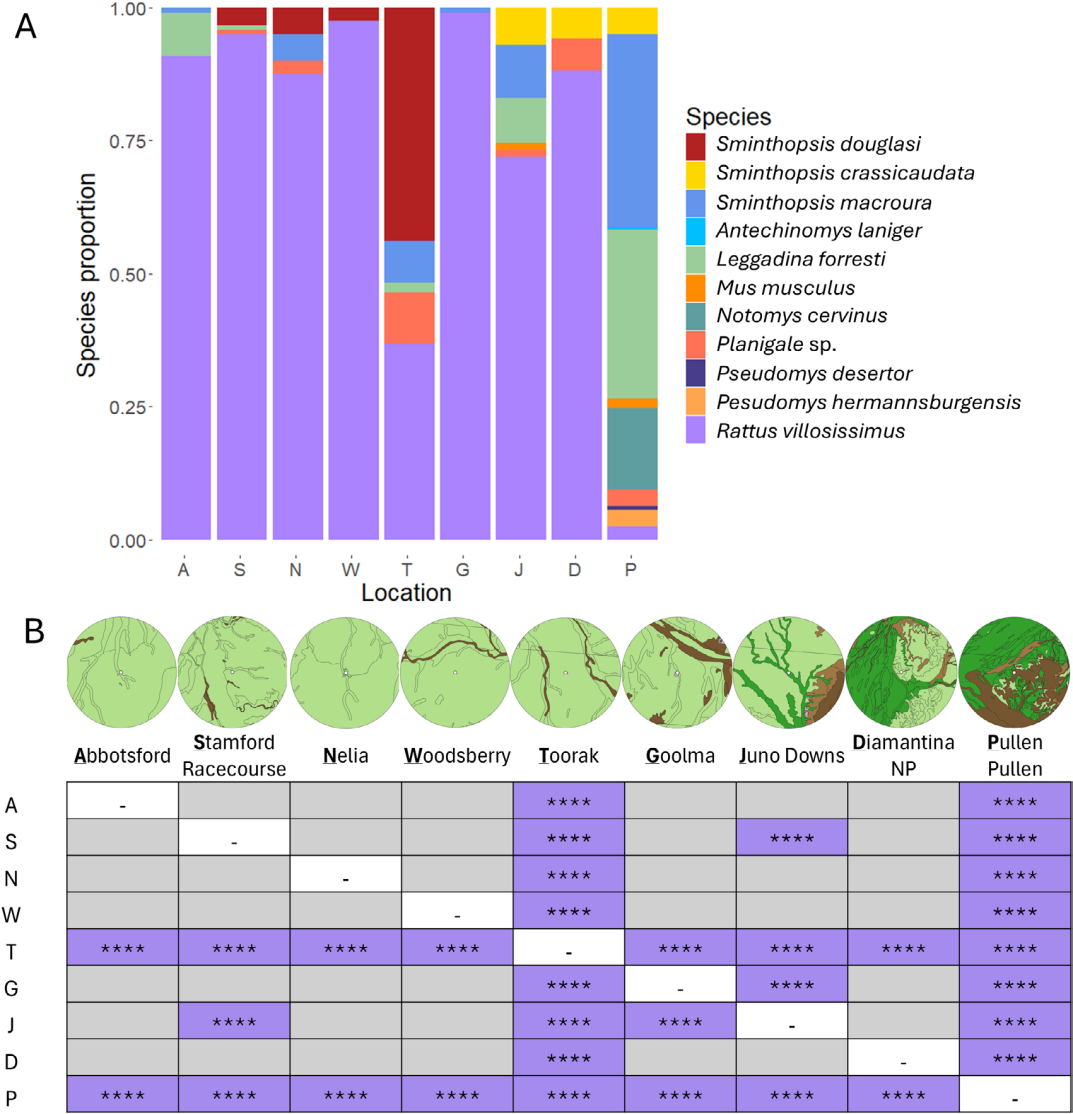
(A) Proportion of each small mammal species at the nine assessed sites. (B) Significance of Fisher's Exact Tests after Bonferroni adjustments (represented by the number of * with the maximum being *p* < 0.0005) between each pair of sites, with the corresponding owl hunting ranges above (10 km radius). Each side of the diagonal is a reflection of the other side.

A maximum of two *Sminthopsis* species were present at each location from the predicted possible combinations of *S. douglasi* , *S. crassicaudata* , and *S. macroura* (Figure 8A). Toorak had significantly different proportions of small mammals in the owl pellets than all other sites, driven primarily by the relatively high proportion of *S. douglasi* (33.1%; *p* < 0.0005) (Figure 8B). Pullen Pullen Reserve was also significantly different from all other sites, driven by the relatively low proportion of *R. villosissimus* (2.1%; *p* < 0.0005). Juno Downs was significantly different to Stamford Racecourse (*p* = 4.95 × 10^−9^) and Goolma (*p* = 5.36 × 10^−9^) (Figure 8). All other pairwise comparisons were not significant.

The location with the largest Shannon's Diversity Index (*H*), when considering all species, was Toorak (1.76), with a species richness of 11, followed closely by Pullen Pullen Reserve (1.72), with a species richness of 13 (Table 2). The location with the largest *H* when considering only mammal species was Pullen Pullen (1.50), and Toorak was second (1.23). Juno Downs had the third largest *H* in both scenarios (all species; mammals only [1.59; 0.98]), with a species richness of 10 in total, including six mammals. The location with the smallest *H* in both cases was Goolma (0.0534; 0.0534) because only two species were found, both mammals. Goolma was also the site with the lowest evenness score (0.077). In both scenarios, Toorak had the highest evenness score (0.732; 0.762), followed by Juno Downs (0.689) when considering all species and Pullen Pullen when only mammals were considered (0.681).

**TABLE 2:** Shannon's Diversity Index (*H*), evenness and species richness of owl pellet contents from each of the nine sites.

*H*: All	A	S	N	W	T	G	J	D	P
Index	1.04	0.722	0.809	0.115	1.76	0.0534	1.59	1.04	1.72
Evenness	0.534	0.347	0.396	0.165	0.732	0.077	0.689	0.645	0.671
Richness	7	8	6	2	11	2	10	5	13
Individuals	141	135	42	41	143	106	137	24	162

*H*: Mammals	A	S	N	W	T	G	J	D	P
Index	0.339	0.243	0.509	0.115	1.23	0.0534	0.982	0.444	1.5
Evenness	0.308	0.176	0.367	0.165	0.762	0.077	0.548	0.404	0.681
Richness	3	4	4	2	5	2	6	3	9
Individuals	98	119	40	41	114	106	71	17	150

Abbreviations: “All”, refers to mammals and birds; A, Abbotsford; D, Diamantina National Park; G, Goolma; J, Juno Downs; N, Nelia; P, Pullen Pullen Reserve; S, Stamford Racecourse; T, Toorak; W, Woodberry.

## 4 Discussion

The primary aim of the present study was to provide presence/absence information for *S. douglasi* at a range of sites with varying probabilities of occurrence across central‐west and north‐west Queensland using owl pellet analysis. This work further highlights the utility of owl pellet analysis in targeting presence monitoring for small mammals (Debus et al. 2008; Woolley 2010; McDonald et al. 2014; Battisti et al. 2019, 2021; Kutt et al. 2020; Drebet 2022). The present study also aimed to broadly examine the habitat category differences between each site in reference to the predicted likelihood of *S. douglasi* occurrence. The present study represents the first large‐scale assessment of *S. douglasi* presence using *T. javanica delicatula* pellet analysis since Woolley's (2010) assessment, which was based on owl pellets collected between 1992 and 2001. *Sminthopsis douglasi* was present at four of the nine locations analyzed, with two sites having historical records of the species (Nelia and Toorak; Woolley 1992; Mifsud 1999; Woolley 2010; Charley et al. 2025a) and two novel sites without a previous record of the species (Stamford Racecourse and Woodsberry).

All four locations with *S. douglasi* presence had a high proportion of grassland habitat within the owl's presumed hunting range (10 km radius of the roost; Hyem 1979). This aligns with our limited knowledge of *S. douglasi* habitat requirements, which are *Astrebla* spp. and *Iseilema* spp. grasslands over cracking clay soils (Mifsud 1999; Kutt 2003; Department of Climate Change, Energy, the Environment and Water 2008; Department of Environment and Resource Management 2009; Waudby and Petit 2017; Woolley 2017). Overall, the results support the accuracy of the Australian Government's habitat distribution models of *S. douglasi* (Department of Climate Change, Energy, the Environment and Water 2024a) as the species was present at all high‐likelihood locations, half the medium‐likelihood locations, and none of the low‐likelihood locations.

Of the low‐likelihood sites, Pullen Pullen Reserve and Diamantina National Park habitats significantly differed from all other sites based on the high proportion of woodland and low proportion of grassland. The limited grassland in proximity to the owl roost may explain why *S. douglasi* was not found on the properties. Alternatively, these sites may represent a distribution limit, with Pullen Pullen, Diamantina National Park, and Juno Downs (low‐likelihood locations) falling just outside of the *S. douglasi* ideal climate envelope (Department of Climate Change, Energy, the Environment and Water 2024a). The northern sites experience more annual rainfall on average (Stamford: 450.1 mm; Julia Creek: 428.2 mm; Winton: 327.3 mm) than the southern sites (Jundah: 323 mm; Diamantina National Park and Pullen Pullen: 235 mm; Bureau of Meteorology 2024). Based on rainfall, Diamantina National Park and Pullen Pullen are arid zone locations (Holzapfel 2008; Department of Climate Change, Energy, the Environment and Water 2024b), which may limit the distribution of this dasyurid that mostly occurs in areas experiencing tropical summer rainfall patterns (Baker and Gynther 2023). However, other habitat characteristics, such as edaphic attributes, may also influence the presence or absence of *S. douglasi* . Edaphic characteristics have been shown to influence the presence of shrew species in Italy in an owl pellet study (Pelosi et al. 2024). This supports the need for further investigation into the potential preference for different soil types by *S. douglasi* .

In previous studies, generic differences in the clay soils have been observed at sites where *S. douglasi* was present or absent. Mifsud (1999, 2001b) noted that *S. douglasi* appears to prefer ashy rather than pebbly clay soils (although it was found on both) as the former are characterized by larger and more frequent cracks (Orr 1975). Such soil characteristics may be important to the species' biology, physiology, and behavior (Mifsud 2001a, 2001c; Department of Environment and Resource Management 2009; Waudby and Petit 2017; Woolley 2017). *Sminthopsis douglasi* uses cracks and holes in the soil to regulate their temperature and water content (Mifsud 2001a; Waudby and Petit 2017), escape from predators (Mifsud 1999) and shelter from fires (Mifsud 1999, 2001b). It is possible that southern, low‐likelihood sites (Diamantina National Park, Pullen Pullen Reserve, and Juno Downs) supported pebbly rather than ashy clay soils, and this warrants further investigation of the microhabitat.

The known southern limit of the species is Bladensburg National Park, but few studies have assessed beyond this point. Woolley (2010) performed an owl pellet analysis assessment of 28 sites (sampled between 1992 and 2001); however, she did not evaluate locations further south than Penola Downs (21.516 S, 141.450 E), located approximately 40 km west of Kynuna. Kutt (2003) surveyed further south at Dunblane (23.450 S, 145.196 E), finding a hair sample from cat guts tentatively identified as *S. douglasi* , but follow‐up trapping detected no individuals. Mifsud (2001b) performed the southernmost survey for *S. douglasi* at Lochern National Park (24.237 S, 143.326 E), also finding no individuals. Of the low‐likelihood sites examined in the present study south of Bladensburg National Park, it is noteworthy that the total sample collected from Pullen Pullen Reserve (700 pellets; Kearney et al. 2022; Schoenefuss 2023) yielded no *S. douglasi* . Other studies performed at Diamantina National Park between 1999 and 2008 also found no *S. douglasi* (Debus et al. 1999, 2008; Palmer 2001; Debus and Rose 2007). Taken together, the evidence suggests *S. douglasi* is not present at Diamantina National Park or Pullen Pullen. However, it would be valuable to assess owl roosts (if they can be located) to the north and east of these sites, falling in predicted medium‐likelihood habitat for the species.

Surprisingly, *S. douglasi* was not found at Abbotsford (100 pellets), even though it was the site with the highest proportion of grassland. Abbotsford's habitat categories were not significantly different from locations where *S. douglasi* was present. The property was mapped to have 100% grassland within a 10 km radius of the owl roost and bordered Stamford Racecourse, where the species was found. Rarefaction showed that the mammal species richness for the location plateaued after the 100‐pellet sample size, suggesting that all species had been detected, which should have included *S. douglasi* if present. However, the probability of detection curves from the four locations where *S. douglasi* were detected indicated that anywhere from 10 to 125 pellets were required to detect the species in this dataset (*pn* > 0.99). Presumably, 10 pellets from a location with a very high abundance of *S. douglasi* and 125 from a location with a lower abundance. Furthermore, confident detection of *S. douglasi* at Toorak was consistently achieved within just 7–10 pellets (*pn* > 0.95) under varying mammal community structures in the owl pellet study of Charley et al. (2025a), a finding corroborated by the present study (10 pellets, *pn* > 0.99). However, when they analyzed the probability of detection at each of Woolley's (2010) locations, Eureka required 161 pellets to reach *pn* > 0.95. Taken together, it is plausible that a small population of *S. douglasi* is present at Abbotsford that was missed with a 100‐pellet sample size, and subsequent collections would allow this to be tested. The present study endorses the recommendation of Charley et al. (2025a) that a minimum of ~200 pellets should be appropriate to detect *S. douglasi* regardless of their relative abundance in the small mammal community.

Another possibility is that the species is indeed absent at Abbotsford because the grassland habitat at this property is not as suitable for *S. douglasi* . Abbotsford has a high cover of prickly acacia ( *Vachellia nilotica* ) (Emma Gray, personal communication, October 25, 2024), which is an invasive weed with root systems that impede soil cracking and is capable of transforming grassland ecosystems into woodland systems (McAlpine and Howes 2005; Department of Environment and Resource Management 2009). Prickly acacia may be negatively associated with *S. douglasi* presence, but this has not been verified (Department of Environment and Resource Management 2009; Smith et al. 2007). *Vachellia nilotica* cannot be reliably detected by satellite images used for regional ecosystem habitat mapping (Neldner et al. 2023), so Abbotsford is still categorized as majority grassland even though *V. nilotica* is found on the property. On‐ground assessments of habitat neighboring the owl roosts are the next logical step to more clearly understand whether it is limiting *S. douglasi* occurrence.

The secondary aim of the present study was to identify differences in the small mammal community across the surveyed sites, focusing on their significance to the owl diet, the relative abundance of species, and the potential interactions with the presence of *S. douglasi* . The most important prey item for *T. javanica delicatula* from eight of the nine sites was *R. villosissimus*. This native rodent species is known to be a key prey item for barn owls (Valente 1981; Debus et al. 1999; Heywood and Pavey 2002; Woolley 2010; Charley et al. 2025a). As volume was a factor in the IRI calculations, both the higher body weight and abundance of this small mammal species are important considerations. From mid‐2023 to at least mid‐late 2024, there was a plague of *R. villosissimus* in northwest Queensland (ABC News 2023, 2024), and evidence of the plague was reflected in a parallel owl pellet study undertaken at Toorak (Charley et al. 2025a). There was also a historical irruption of *R. villosissimus* at Bladensburg National Park in 2009, according to Baker (2013, personal communication with Mifsud). The timing aligns with the fact that in the present study, Juno Downs (collected 2009) also had *R. villosissimus* as the most important prey item in barn owl pellets.

In contrast, at Pullen Pullen Reserve (collected 2019), the dasyurid *S. macroura* was the most important prey item. Similarly, live‐trapping (metal box and pitfall) surveys conducted simultaneously (2018 and 2019) by Kearney et al. (2020) at Pullen Pullen found that the most prevalent mammal species was *S. macroura* . There were no reported rodent irruptions at this time, which may partly explain why *R. villosissimus* comprised only 1.2% of the IRI from this location. However, the higher proportion of woodland at this site may also be a factor. Newsome and Corbett (1975) found that in the absence of a plague, *R. villosissimus* was present in semi‐arid and arid grassland and desert habitats but not in acacia woodlands. Furthermore, *S. macroura* may be present in higher proportions at Pullen Pullen due to the diversity of habitat categories (grassland, herbland, shrubland and woodland). Frank and Soderquist (2005) suggested that a greater diversity and coverage of vegetation may provide a higher biomass of arthropods for the *S. macroura* carnivorous diet. Additionally, higher shrub cover may protect the species from predators, and the absence of livestock, as is the case on the Pullen Pullen Reserve, would lead to minimal soil compaction that may otherwise compromise essential soil cracks used by small mammals for refuge (Frank and Soderquist 2005).

Assessment of the total small mammal community indicated that Toorak and Pullen Pullen were significantly different to all other sites. This was primarily driven by Toorak's relatively high proportion of *S. douglasi* , and Pullen Pullen's low proportion of *R. villosissimus* and relatively high proportions of *S. macroura* and the mouse, *L. forresti*. Most likely, this is reflective of the abundance of these prey species as proportions of the total small mammal community (Yom‐Tov and Wool 1997; Heywood and Pavey 2002; Andrade et al. 2016; Schoenefuss et al. 2024; Charley et al. 2025a).

Toorak was identified as a key location for the long‐term persistence of *S. douglasi* in the national recovery plan (Department of Environment and Resource Management 2009), a finding reported by Charley et al. (2025a) and corroborated in the present study. While the data suggest that a high proportion of grassland may predict the presence of *S. douglasi* , there is an opportunity to further assess Toorak and its microhabitat features through on‐ground surveys.

Interestingly, when *Sminthopsis* was found on a property, a maximum of two species were present from the predicted combinations of *S. douglasi* , *S. crassicaudata* , and *S. macroura* . It is plausible that all three species rarely co‐occur. Woolley (2010) only found one or both of *S. douglasi* and *S. macroura* at all 28 assessed sites, with no *S. crassicaudata* present in the owl pellets, a finding corroborated at Toorak by Charley et al. (2025a), which analyzed 2 years of data (2023 and 2024). Similarly, Schoenefuss (2023) found both *S. macroura* and *S. crassicaudata* at Pullen Pullen Reserve. All three species were cumulatively present on cameras distributed across 10 sites in the grassland habitat of northern Bladensburg National Park (Tighe 2022); however, no more than two species were observed at the same camera site. Further investigations into the relative occurrence of the three *Sminthopsis* should be undertaken, particularly given that investigating the interactions between sympatric species of small mammals is a recommended action in the *S. douglasi* national recovery plan (Department of Environment and Resource Management 2009).

Incidence rarefaction suggested that a sample size of 100 owl pellets was adequate for Abbotsford, Toorak, Goolma, and Juno Downs to detect all species. Conversely, the small mammal species richness at Stamford Racecourse, Diamantina National Park, Nelia, and Woodsberry was predicted to increase with a larger sample size. Juno Downs species richness was predicted to rise with a larger sample size of individuals, indicating that this site too may need to be resampled. Diamantina has been a site of owl pellet analysis in the past, and *S. douglasi* was not detected (101 pellets collected between 1998 and 2006; Debus et al. 1999; Palmer 2001; Debus and Rose 2007). Nelia has also been previously assessed with owl pellet analysis; Woolley (2010) found seven individuals in 142 pellets (collected between 1994 and 1996), and *S. douglasi* presence at this site was corroborated in the present study. However, Woodsberry, Stamford Racecourse, and Juno Downs are newly studied locations that would benefit from repeated pellet sampling.

Overall, the owl pellet analysis detection technique utilized in the present study was effective. The proportion of *S. douglasi* from the total number of prey items analyzed in the present study ranged from 2.3% to 33.1%. In comparison, live trapping studies targeting *S. douglasi* have had generally lower success rates, with the highest (6.75%) recorded by Mifsud (2001a) at Bladensburg National Park. In contrast, Baker (2013) only found one individual *S. douglasi* at Bladensburg after 5400 trap nights (0.019%). In their recent trapping study of *S. douglasi* , Bakker, Patterson, et al. (2024) recorded success rates ranging from 0% to 2.14% at Bladensburg National Park, capturing 49 individuals over 7798 trap nights. Mifsud (1999) found only a single *S. douglasi* individual after 17,300 trap nights of effort at Toorak in 1995. The following year, no individuals were caught after 4500 trap nights despite their known presence on the property (Mifsud 1999). This is striking given the high proportion of *S. douglasi* present in the owl pellets from Toorak (Charley et al. 2025a; present study) and the relatively small owl pellet collections (*n* ≤ 100) from the present study that contained *S. douglasi* (Nelia, Woodsberry and Stamford Racecourse). The amount of labor, money, and time needed to perform live trapping at remote arid locations, like those within the *S. douglasi* range, greatly exceeds that needed for owl pellet analysis (McDonald et al. 2014; Drebet 2022; Schoenefuss 2023). The trade‐off remains the type of data that can be obtained from each method. If an owl roost can be located, and the primary aim is to determine the presence/absence of a small mammal (rather than measurements and growth data requiring a live animal), then owl pellet analysis is a most viable technique.

## Author Contributions

**Dana A. Lockhart:** conceptualization (equal), data curation (lead), formal analysis (lead), investigation (lead), methodology (lead), visualization (lead), writing – original draft (lead). **Joshua J. Bon:** formal analysis (supporting), writing – review and editing (supporting). **Cameron L. Charley:** data curation (supporting), writing – review and editing (supporting). **Stephen G. Kearney:** data curation (supporting), writing – review and editing (supporting). **Pia Schoenefuss:** data curation (supporting), writing – review and editing (supporting). **Emma L. Gray:** conceptualization (equal), formal analysis (supporting), methodology (supporting), project administration (supporting), supervision (supporting), writing – review and editing (supporting). **Andrew M. Baker:** conceptualization (equal), funding acquisition (lead), methodology (supporting), project administration (lead), supervision (lead), writing – review and editing (supporting).

## Conflicts of Interest

The authors declare no conflicts of interest.

## Supporting information


**Appendix S1.** Index of relative importance (IRI) from each of the nine owl roost locations displaying the factors incorporated into the IRI calculations ((Numerical percentage + volumetric percentage) × frequency of occurrence percentage). No: Number; Freq: Frequency.

## Data Availability

The authors confirm that the data supporting the findings of this study are available within the article [and/or] its Supporting Information. Data are available via https://doi.org/10.5281/zenodo.14886441.
