# Supplementary material for: The Hidden Diet: Determining the Distribution of the Threatened Julia Creek Dunnart (Sminthopsis douglasi) Using Eastern Barn Owl (Tyto javanica delicatula) Pellets
Source: Ecol Evol. 2025 Jun 27;15(7):e71617. doi: 10.1002/ece3.71617 (PMC12203013; doi:10.1002/ece3.71617)
Supplement: Supplementary file 1 — Appendix S1. Index of relative importance (IRI) from each of the nine owl roost locations displaying the factors incorporated into the IRI calculations ((Numerical percentage + volumetric percentage) × frequency of occurrence percentage). No: Number; Freq: Frequency. [file ECE3-15-e71617-s001.docx]

Appendix 1: Index of relative importance (IRI) from each of the nine owl roost locations displaying the factors incorporated into the IRI calculations ((Numerical percentage + volumetric percentage) x frequency of occurrence percentage). No: Number; Freq: Frequency.

| **Prey** | **No** | **No** | **Freq** | **Weight** | **Volume** | **IRI** |
| --- | --- | --- | --- | --- | --- | --- |
|  |  | **(%)** | **(%)** |  | **(%)** | **(rank; %)** |
| **Abbotsford (100 pellets)** |  |  |  |  |  |  |
| Invertebrates | n.a. | n.a. | 3.0 | n.a. | n.a. | n.a. |
| Birds |  |  |  |  |  |  |
| *Aves* sp. | 4 | 2.6 | 3.0 | n.a. | n.a. | n.a. |
| *Taeniopygia* sp. | 1 | 0.7 | 1.0 | 12 | 0.1 | 0.7 (7; **<0.1**) |
| *Artamus* sp. | 6 | 3.9 | 6.0 | 40 | 1.8 | 34.4 (4; **0.3**) |
| *Melopsittacus* | 35 | 22.9 | 18.0 | 35 | 9.2 | 578 (2; **5.8**) |
| *undulatus* |  |  |  |  |  |  |
| Mammals |  |  |  |  |  |  |
| *Rattus villosissimus* | 89 | 58.2 | 64.0 | 130 | 87.2 | 9304.2 (1; **93.4**) |
| *Leggadina forresti* | 8 | 5.2 | 7.0 | 20 | 1.2 | 45.0 (3; **0.5**) |
| *Sminthopsis macroura* | 1 | 0.7 | 1.0 | 20 | 0.2 | 0.8 (6; **<0.1**) |
| *Sminthopsis* sp. | 2 | 1.3 | 2.0 | 17.5 | 0.3 | 3.1 (5; **<0.1**) |
| Reptiles |  |  |  |  |  |  |
| Squamata sp*.* (lizards) | 1 | 0.7 | 1.0 | 5 | 0.0 | 0.7 (8; **<0.1**) |
| Unidentifiable/ nothing | 6 | 3.9 | 6.0 | n.a. | n.a. | n.a. |
| **Diamantina National Park (24 pellets)** | | | |  |  |  |
| Invertebrates | n.a. | n.a. | 29.2 | n.a. | n.a. | n.a. |
| Birds |  |  |  |  |  |  |
| *Aves spp.* | 1 | 3.6 | 4.2 | n.a. | n.a. | n.a. |
| *Taeniopygia* sp. | 6 | 21.4 | 12.5 | 12 | 3.5 | 311.8 (2; **3.2**) |
| Mammals |  |  |  |  |  |  |
| *Rattus villosissimus* | 15 | 53.6 | 62.5 | 130 | 95.3 | 9302.0 (1; **96.3**) |
| *Sminthopsis* | 1 | 3.6 | 4.2 | 15 | 0.7 | 17.9 (3; **0.2**) |
| *crassicaudata* |  |  |  |  |  |  |
| *Planigale* sp. | 1 | 3.6 | 4.2 | 5 | 0.2 | 15.9 (4; **0.2**) |
| Reptiles |  |  |  |  |  |  |
| Squamata sp. (lizards) | 1 | 3.6 | 4.2 | 5 | 0.2 | 15.9 (4; **0.2**) |
| Unidentifiable/ nothing | 3 | 0.1 | 12.5 | n.a. | n.a. | n.a. |
| **Goolma (100 pellets)** |  |  |  |  |  |  |
| Invertebrates | n.a. | n.a. | 4.0 | n.a. | n.a. | n.a. |
| Mammals |  |  |  |  |  |  |
| *Rattus villosissimus* | 105 | 92.1 | 92.0 | 130.0 | 99.9 | 17660.2 (1; **>99.9**) |
| *Sminthopsis macroura* | 1 | 0.9 | 1.0 | 20.0 | 0.1 | 1.0 (2; **<0.1**) |
| Unidentifiable/ nothing | 8 | 7.0 | 8.0 | n.a. | n.a. | n.a. |

| **Prey** | **No** | **No** | **Freq** | **Weight** | **Volume** | **IRI** |
| --- | --- | --- | --- | --- | --- | --- |
|  |  | **(%)** | **(%)** |  | **(%)** | **(rank; %)** |
| **Juno Downs (100 pellets)** |  |  |  |  |  |  |
| Invertebrates | n.a. | n.a. | 22.0 | n.a. | n.a. | n.a. |
| Birds |  |  |  |  |  |  |
| *Aves* spp*.* | 8 | 5.2 | 7.0 | n.a. | n.a. | n.a. |
| *Artamus* sp. | 16 | 10.3 | 15.0 | 40.0 | 6.8 | 257.2 (3; **5.1**) |
| *Melopsittacus* | 46 | 29.7 | 31.0 | 35.0 | 17.2 | 1451.9 (2; **28.9**) |
| *undulatus* |  |  |  |  |  |  |
| *Turnix* sp. | 1 | 0.6 | 1.0 | 70.0 | 0.7 | 1.4 (9; **<0.1**) |
| Mammals |  |  |  |  |  |  |
| *Rattus villosissimus* | 51 | 32.9 | 31.0 | 130.0 | 70.7 | 3210.6 (1; **63.9**) |
| *Leggadina forresti* | 6 | 3.9 | 5.0 | 20.0 | 1.3 | 25.7 (5; **0.5**) |
| *Sminthopsis macroura* | 7 | 4.5 | 7.0 | 20.0 | 1.5 | 42.1 (4; **0.8**) |
| *Sminthopsis* | 5 | 3.2 | 5.0 | 15.0 | 0.8 | 20.1 (6; **0.4**) |
| *crassicaudata* |  |  |  |  |  |  |
| *Sminthopsis* spp. | 3 | 1.9 | 3.0 | 17.5 | 0.6 | 7.5 (7; **0.1**) |
| *Planigale* sp*.* | 1 | 0.6 | 1.0 | 5.0 | 0.1 | 0.7 (11; **<0.1**) |
| *Mus musculus* | 1 | 0.6 | 1.0 | 25.0 | 0.3 | 0.9 (10; **<0.1**) |
| Reptiles |  |  |  |  |  |  |
| Squamata spp. | 3 | 1.9 | 2.0 | 5.0 | 0.2 | 4.2 (8; **0.1**) |
| (lizards) |  |  |  |  |  |  |
| Unidentifiable/ nothing | 7 | 4.5 | 7.0 | n.a. | n.a. | n.a. |
| **Nelia (40 pellets)** |  |  |  |  |  |  |
| Invertebrates | n.a. | n.a. | 12.5 | n.a. | n.a. | n.a. |
| Birds |  |  |  |  |  |  |
| *Aves* spp. | 6 | 11.3 | 7.5 | n.a. | n.a. | n.a. |
| *Mirafra javanica* | 1 | 2.4 | 2.5 | 22 | 0.5 | 6.6 (4; **0.1**) |
| *Melopsittacus* | 1 | 2.4 | 2.5 | 35 | 0.7 | 5.9 (5; **0.1**) |
| *undulatus* |  |  |  |  |  |  |
| Mammals |  |  |  |  |  |  |
| *Rattus villosissimus* | 35 | 83.3 | 70.0 | 130 | 95.5 | 11311.0 (1; **99.4**) |
| *Sminthopsis douglasi* | 2 | 4.8 | 5.0 | 55 | 2.3 | 30.4 (2; **0.3**) |
| *Sminthopsis macroura* | 2 | 4.8 | 5.0 | 20 | 0.8 | 23.1 (3; **0.2**) |
| *Planigale* sp. | 1 | 2.4 | 2.5 | 5 | 0.1 | 5.0 (6; **<0.1**) |
| Unidentifiable/ nothing | 5 | 9.4 | 12.5 | n.a. | n.a. | n.a. |

| **Prey** | **No** | **No** | **Freq** | **Weight** | **Volume** | **IRI** |
| --- | --- | --- | --- | --- | --- | --- |
|  |  | **(%)** | **(%)** |  | **(%)** | **(rank; %)** |
| **Pullen Pullen (100 pellets)** |  |  |  |  |  |  |
| Invertebrates | n.a. | n.a. | 53.0 | n.a. | n.a. | n.a. |
| Birds |  |  |  |  |  |  |
| *Taeniopygia* sp. | 1 | 0.5 | 6.0 | 12.0 | 0.3 | 4.8 (12; **0.1**) |
| *Melopsittacus* | 2 | 1.0 | 2.0 | 35.0 | 7.0 | 16.0 (7; **0.3**) |
| *undulatus* |  |  |  |  |  |  |
| *Aves* spp. | 8 | 4.2 | 8.0 | n.a. | n.a. | n.a. |
| Mammals |  |  |  |  |  |  |
| *Sminthopsis macroura* | 57 | 29.8 | 40.0 | 20.0 | 26.1 | 2237.1 (1; **46.0**) |
| *Planigale tenuirostris* | 5 | 2.6 | 5.0 | 5.0 | 0.6 | 15.9 (8; **0.3**) |
| *Notomys cervinus* | 22 | 11.5 | 20.0 | 35.0 | 17.6 | 582.7 (3; **12.0**) |
| Rodent spp. | 5 | 2.6 | 4.0 | n.a. | n.a. | n.a. |
| *Rattus villosissimus* | 4 | 2.1 | 4.0 | 134.0 | 12.3 | 57.4 (4; **1.2**) |
| *Leggadina forresti* | 50 | 26.2 | 37.0 | 20.0 | 22.9 | 1815.2 (2; **37.3**) |
| *Nototmys cf. cervinus* | 2 | 1.0 | 1.0 | 35.0 | 1.6 | 2.6 (14; **2.6**) |
| *Notomys* sp. | 5 | 2.6 | 4.0 | 67.5 | 7.7 | 41.4 (6; **0.9**) |
| *Psuedomys* sp. | 2 | 1.0 | 2.0 | 25.0 | 1.1 | 4.4 (13; **0.1**) |
| *Pseudomys* | 5 | 2.6 | 3.0 | 12.0 | 1.4 | 12.0 (10; **0.2**) |
| *hermannsburgensis* |  |  |  |  |  |  |
| *Sminthopsis* spp. | 4 | 2.1 | 4.0 | 17.5 | 1.6 | 14.8 (9; **0.3**) |
| *Mus musculus* | 3 | 1.6 | 2.0 | 20.0 | 1.4 | 5.9 (11; **0.1**) |
| *Sminthopsis* | 8 | 4.2 | 7.0 | 15.0 | 2.7 | 48.5 (5; **1.0**) |
| *crassicaudata* |  |  |  |  |  |  |
| *Pseudomys desertor* | 1 | 0.5 | 1.0 | 25.0 | 0.6 | 1.1 (15; **<0.1**) |
| *Antechinomys laniger* | 1 | 0.5 | 1.0 | 25.0 | 0.6 | 1.1 (15; **<0.1**) |
| Amphibians |  |  |  |  |  |  |
| Amphibia spp. | 2 | 1.0 | 2.0 | n.a. | n.a. | n.a. |
| Unidentifiable/ nothing | 4 | 2.1 | 4.0 | n.a. | n.a. | n.a. |

| **Prey** | **No** | **No** | **Freq** | **Weight** | **Volume** | **IRI** |
| --- | --- | --- | --- | --- | --- | --- |
|  |  | **(%)** | **(%)** |  | **(%)** | **(rank; %)** |
| **Stamford Racecourse  (100 pellets)** |  |  |  |  |  |  |
| Invertebrates | n.a. | n.a. | 13.0 | n.a. | n.a. | n.a. |
| Birds |  |  |  |  |  |  |
| *Aves* spp. | 2 | 1.4 | 1.0 | n.a. | n.a. | n.a. |
| *Taeniopygia* sp. | 7 | 5.0 | 3.0 | 12.0 | 0.5 | 16.6 (4; **0.1**) |
| *Melopsittacus* | 3 | 2.1 | 2.0 | 35.0 | 0.7 | 5.7 (5; **<0.1**) |
| *undulatus* |  |  |  |  |  |  |
| *Artamus* sp. | 5 | 3.6 | 4.0 | 40.0 | 1.3 | 19.5 (2; **0.1**) |
| Mammals |  |  |  |  |  |  |
| *Rattus villosissimus* | 113 | 80.7 | 90.0 | 130.0 | 95.8 | 15359.5 (1; **99.6**) |
| *Sminthopsis douglasi* | 4 | 2.9 | 4.0 | 55.0 | 1.4 | 17.2 (3; **0.1**) |
| *Leggadina forresti* | 1 | 0.7 | 1.0 | 20.0 | 0.1 | 0.8 (6; **<0.1**) |
| *Planigale* sp. | 1 | 0.7 | 1.0 | 5.0 | 0.0 | 0.7 (7; **<0.1**) |
| Reptiles |  |  |  |  |  |  |
| Squamata sp. (lizards) | 1 | 0.7 | 1.0 | 5.0 | 0.0 | 0.7 (7; **<0.1**) |
| Unidentifiable/ nothing | 3 | 2.1 | 3.0 | n.a. | n.a. | n.a. |
| **Woodsberry (31 pellets)** |  |  |  |  |  |  |
| Invertebrates | n.a. | n.a. | 9.7 | n.a. | n.a. | n.a. |
| Mammals |  |  |  |  |  |  |
| *Rattus villosissimus* | 40 | 93.0 | 93.5 | 130 | 99.0 | 861109.7 (1; **>99.9**) |
| *Sminthopsis douglasi* | 1 | 2.3 | 3.2 | 55 | 1.0 | 10.9 (2; **<0.1**) |
| Unidentifiable/ nothing | 2 | 4.7 | 0.1 | n.a. | n.a. | n.a. |
| **Toorak (100 pellets)** |  |  |  |  |  |  |
| Birds |  |  |  |  |  |  |
| *Artamus* sp. | 2 | 1.3 | 2 | 40 | 0.9 | 4.4 (7; **0.1**) |
| *Taeoniopygia* sp. | 15 | 9.9 | 7 | 12 | 2 | 83.5 (3; **1.4**) |
| *Mirafra javanica* | 7 | 4.6 | 6 | 22 | 1.7 | 38.1 (6; **0.6**) |
| *Aves* spp. | 6 | 4.0 | 4 | n.a. | n.a. | n.a. |
| *Melopsittacus* | 2 | 1.3 | 2 | 35 | 0.8 | 4.2 (8; **0.1**) |
| *undulatus* |  |  |  |  |  |  |
| *Anthus australis* | 1 | 0.7 | 1 | 24 | 0.3 | 0.9 (10; **<0.1**) |
| Mammals |  |  |  |  |  |  |
| *Sminthopsis douglasi* | 50 | 33.1 | 36 | 55 | 30.6 | 2292.9 (2; **38.8**) |
| *Sminthopsis macroura* | 9 | 6.0 | 7 | 20 | 2 | 55.7 (4; **0.9**) |
| *Planigale* spp. | 11 | 7.3 | 7 | 5 | 0.6 | 55.3 (5; **0.9**) |
| *Rattus villosissimus* | 42 | 27.8 | 38 | 130 | 60.7 | 3364.1 (1; **57.0**) |
| *Leggadina forresti* | 2 | 1.3 | 2 | 20 | 0.4 | 3.5 (9; **0.1**) |
| Amphibians |  |  |  |  |  |  |
| Amphibia spp. | 2 | 1.3 | 1 | n.a. | n.a. | n.a. |
| Unidentifiable/ nothing | 2 | 1.3 | 2 | n.a. | n.a. | n.a. |
